# Deletion of an African Swine Fever Virus ATP-Dependent RNA Helicase *QP509L* from the Highly Virulent Georgia 2010 Strain Does Not Affect Replication or Virulence

**DOI:** 10.3390/v14112548

**Published:** 2022-11-17

**Authors:** Elizabeth Ramirez-Medina, Elisabeth A. Vuono, Sarah Pruitt, Ayushi Rai, Nallely Espinoza, Edward Spinard, Alyssa Valladares, Ediane Silva, Lauro Velazquez-Salinas, Manuel V. Borca, Douglas P. Gladue

**Affiliations:** 1 Plum Island Animal Disease Center, Agricultural Research Service, United States Department of Agriculture, Greenport, USDA, Greenport, NY 11944, USA; 2 Department of Pathobiology and Population Medicine, Mississippi State University, Starkville, MS 39762, USA; 3 Oak Ridge Institute for Science and Education (ORISE), Oak Ridge, TN 37830, USA; 4 Department of Anatomy and Physiology, Kansas State University, Manhattan, KS 66506, USA

**Keywords:** ASFV, ASF, African swine fever, African swine fever virus, *QP509L*, virus virulence

## Abstract

African swine fever virus (ASFV) produces a lethal disease (ASF) in domestic pigs, which is currently causing a pandemic deteriorating pig production across Eurasia. ASFV is a large and structurally complex virus with a large genome harboring more than 150 genes. ASFV gene *QP509L* has been shown to encode for an ATP-dependent RNA helicase, which appears to be important for efficient virus replication. Here, we report the development of a recombinant virus, ASFV-G-∆QP509L, having deleted the *QP509L* gene in the highly virulent field isolate ASFV Georgia 2010 (ASFV-G). It is shown that ASFV-G-∆QP509L replicates in primary swine macrophage cultures as efficiently as the parental virus ASFV-G. In addition, the experimental inoculation of pigs with 10^2^ HAD_50_ by the intramuscular route produced a slightly protracted but lethal clinical disease when compared to that of animals inoculated with virulent parental ASFV-G. Viremia titers in animals infected with ASFV-G-∆QP509L also had slightly protracted kinetics of presentation. Therefore, ASFV gene *QP509L* is not critical for the processes of virus replication in swine macrophages, nor is it clearly involved in virus replication and virulence in domestic pigs.

## 1. Introduction

African swine fever virus (ASFV) is the causative agent of African swine fever (ASF), an often-lethal disease that affects both wild and domestic pigs [1]. ASF is currently causing a pandemic affecting Africa, Eurasia, and more recently the Hispaniola Island in the Caribbean region, severely damaging swine production. Recently, the first commercial vaccine was approved for use in Vietnam [1]; however, it is not available in other countries. In countries without an approved vaccine, disease control is limited to the isolation of the outbreak and culling all infected animals in the affected geographical area [2].

ASFV is a structurally complex virus possessing a large DNA genome (180–190 Kb) encoding for more than 150 genes [3]. Many of these genes’ functions have not been predicted or defined. The deletion of individual virulence-associated ASFV genes led to the development of recombinant attenuated virus strains that offer protection against ASF when challenged with the homologous genetic backbone, resulting in several experimental live attenuated vaccine candidates [1,4,5,6,7,8,9,10]. Similarly, detecting and characterizing viral genes involved in the process of virus replication may lead to the possible development of therapeutic tools to control or abort virus infection. The ASFV *QP509L* gene has been demonstrated to have RNA helicase activity [11], and siRNA inhibition of the gene transcription provokes a reduction of over 99% in virus yields. In addition, the deletion of the *QP509L* gene from the genome of highly virulent field isolate ASFV CN/GS/2018 produced partial attenuation of the virus virulence in domestic pigs [12]. The additional deletion of an adjacent gene, *QP383R*, produced a complete attenuation of the CN/GS/2018 strain [12].

Here, we show that ASFV-G-∆QP509L, a recombinant virus harboring the deletion of the *QP509L* gene in the genome of the highly virulent Georgia 2010 isolate, efficiently replicates in primary swine macrophage and domestic pigs. The animals infected with ASFV-G-∆QP509L showed a slightly protracted but still lethal clinical disease when compared to that of animals inoculated with virulent parental ASFV-G. Therefore, ASFV gene *QP509L* is not critical for the processes of virus replication in swine macrophages, nor is it involved in virus replication and virulence in domestic pigs.

## 2. Materials and Methods 

### 2.1. Viruses and Cells

Primary swine macrophage cultures were produced from blood macrophages obtained as previously described [13]. Cell culture plates were seeded at a density of 5 × 10^6^ macrophages per mL. ASFV Georgia (ASFV-G) was a field isolate kindly provided by Dr. Nino Vepkhvadze from the Laboratory of the Ministry of Agriculture (LMA) in Tbilisi, Republic of Georgia [14]. ASFV-G-∆QP509L and ASFV g comparative growth curves were performed on primary swine macrophages using six-well plates as previously described [14]. The plates were seeded with a virus MOI of 0.01 HAD_50_ (hemadsorbing doses), and sample points were taken at 2, 24, 48, 72, and 96 h. The cells were frozen at ≤−70 °C, thawed, and the lysates titrated in primary swine macrophage cell cultures in 96-well plates. The presence of the virus in infected cells was assessed by hemadsorption (HA), and the virus titers were calculated as previously described [15].

### 2.2. Detection of QP509L Transcription

A real-time PCR assay (qPCR) was used to evaluate the transcriptional profile of the *QP509L* gene during the infection of ASFV g in cultures of porcine macrophages, as previously described [16]. The detection of the expression of the *CP204L* (p30) and *B646L* (p72) was used as early and late transcribed reference genes. Briefly, cell cultures of porcine macrophages were infected with a stock of ASFV g using an MOI of 1. RNA extractions, using an RNeasy Kit (QIAGEN, Hilden, Germany), were conducted at 0, 4, 6, 8, and 24 h post-infection. All extractions were treated with 2 units of DNase I (BioLabs, San Diego, CA, USA), then purified using the Monarch^®^ RNA Cleanup Kit (New England BioLabs, Inc., Ipswich, MA, USA). In total, 1 ug of RNA was used to produce cDNA using qScript cDNA SuperMix (Quanta bio, Beverly, MA, USA) which was used for the qPCR. Primers and probes for the detection of the *QP509L* gene were designed using the ASFV Georgia 2007/1 strain (GenBank Accession #NC_044959.2). Forward Primer: 5′-CACCAAGGCGATTCAAATACAG-3′, reverse primer: 5′-TCGTACTCTTTGCTCGTCATG-3′, and probe: 5-FAM-TAACATTGCTCCTGCCCCACCT-MGBNFQ-3′. Primers and probes for the detection of p72 gene: forward 50-CTTCGGCGAGCGCTTTATCAC-30, reverse: 5′-GGAAATTCATTCACCAAATCCTT-3′, and probe: 5′-FAM-CGATGCAAGCTTTAT -MGB NFQ-3′. Primers and probes for the detection of the CP204L (p30) gene: forward 5′-GACGGAATCCTCAGCATCTTC-3′, reverse: 5′-CAGCTTGGAGTCTTTAGGTACC-3′, and probe: 5′-FAM-TGTTTGAGCAAGAGCCCTCATCGG-MGBNFQ-3′. Primers and probes for the detection of the β-actin gene: forward 5′-GACCTGACCGACTACCTCATG-3′, reverse: 5′-TCTCCTTGATGTCCCGCAC-3′, and probe: 5′-FAM-CTACAGCTTCACCACCACGGC-MGBNFQ-3′. All qPCRs were conducted using the TaqMan Universal PCR Master Mix (Applied Biosystems, Waltham, MA, USA) using the following amplification conditions: one step at 55 °C for 2 min, followed by one denaturation step at 95 °C for 10 min, then 40 cycles of denaturation at 95 °C for 15 s, and annealing/extension at 65 °C for 1 min.

### 2.3. Construction of the ASFV QP509L Deletion Mutant

A recombinant mutant virus was developed to delete the *QP509L* gene, which overlaps on both sides with other ASFV genes. The *QP383R* gene overlaps the N-terminus of the *QP509L* gene by 11 nucleotides. Accordingly, the recombinant vector was designed to conserve these 11 nucleotides along with the 200 bp promoter region of the *QP385R* gene to preserve the functional integrity of the *QP383R* gene. The *QP509L* C-terminus also overlaps the N-terminus of the *Q706L* gene by 43 nucleotides; therefore, the recombination vector was designed to leave these 43 nucleotides and a 200 bp promoter region intact for the *Q706L* gene, preserving 243 nucleotides at the C-terminus of the *QP509L* gene. In order not to interfere with the overlapping genes or their promoter regions, the resulting virus only deleted the central 1075 nucleotides of the *QP509L* gene. ASFV-G-∆QP509L was the result of the homologous recombination of the parental virus (ASFV-G) genome and the recombination transfer vector p72mCherryΔQP509L, following procedures previously described [9]. p72mCherryΔQP509L encompasses the ASFV genomic regions adjacent to the *QP509L* gene: the left region is located between the genomic positions 159,245–160,245, while the right region is located between the genomic positions 161,321–162,321. As already explained, the construct will create a 1075-nucleotide deletion between the nucleotide positions 160,246–161,320. The p72mCherryΔQP509L transfer vector also has a reporter gene cassette containing the mCherry fluorescent protein (mCherry) gene under the control of the ASFV p72 late gene promoter [17]. The p72mCherryΔQP509L vector was obtained by DNA synthesis (Epoch Life Sciences, Sugar Land, TX, USA). The recombinant ASFV-G-∆QP509L was purified by consecutive limiting dilution steps in 96-well plates with individual wells observed and selected based on mCherry detection in individual wells and full-length sequenced using next-generation sequencing (NGS).

### 2.4. Next-Generation Sequencing of ASFV

Virus DNA was extracted from infected macrophage cultures presenting CPE higher than 90%. The nucleus and cytoplasmic fractions were separated using the nuclear extraction kit with the viral DNA isolated from the cytoplasmic fraction, following the manufacturer’s protocol (Active Motif cat# 40,010). ASFV-infected cells were treated with the hypotonic buffer on ice until the cell membrane was dissolved. The nucleus fraction was separated by centrifugation, the cytoplasmic fraction was collected, and DNA was extracted by adding 10% (*v*/*v*) of 3 M Na0Ac (Sigma-Aldrich 71,196, St. Louis, MO, USA) and an equal volume of phenol:chloroform:isoamyl alcohol (25:24:1) with a pH of 6.5–6.9 (Sigma-Aldrich P3803-100 mL, St. Louis, MO, USA). The preparation was centrifuged for maximum speed in a tabletop centrifuge. The aqueous layer was then ethanol precipitated using 2 volumes of 100% ethanol, washed with the same volume of 70% ethanol, and dried. The resulting DNA pellet was then reconstituted in sterile water. We then used this DNA library for NGS sequencing, using a Nextera XT kit in the NextSeq550 (Illumina, San Diego, CA, USA) following the manufacturer’s protocol. Sequence analysis was performed using CLC Genomics Workbench software (CLCBio, Waltham, MA, USA). Briefly, 891,404 reads mapped to ASFV g and a coverage map shows a deletion in QP509L (Supplementary Figure S1).

### 2.5. Evaluation of ASFV-G-ΔQP509L Virulence in Domestic Pigs

The virulence of the ASFV-G-∆QP509L was evaluated in commercial breed pigs. Five animals (weighting approximately 35–40 kg) were intramuscularly (IM) inoculated with 10^2^ HAD_50_ of ASFV-G-∆QP509L while, as control, a similar group of animals was inoculated with 10^2^ HAD_50_ of ASFV-G. The appearance of clinical signs (anorexia, depression, fever, purple skin discoloration, staggering gait, diarrhea, and cough) and body temperature were monitored daily during the experiment. Blood samples were scheduled to be obtained at 0, 4, 7, 11, 14, 21, and 28 days post-inoculation (pi). Animal experiments were performed under biosafety level 3 conditions in the animal facilities at the Plum Island Animal Disease Center, following a strict protocol approved by the Institutional Animal Care and Use Committee (number 225.06-19-R, approved 9 October 2019).

## 3. Results and Discussion

### 3.1. Evolution of the RNA Helicase QP509L

**Figure 1:**
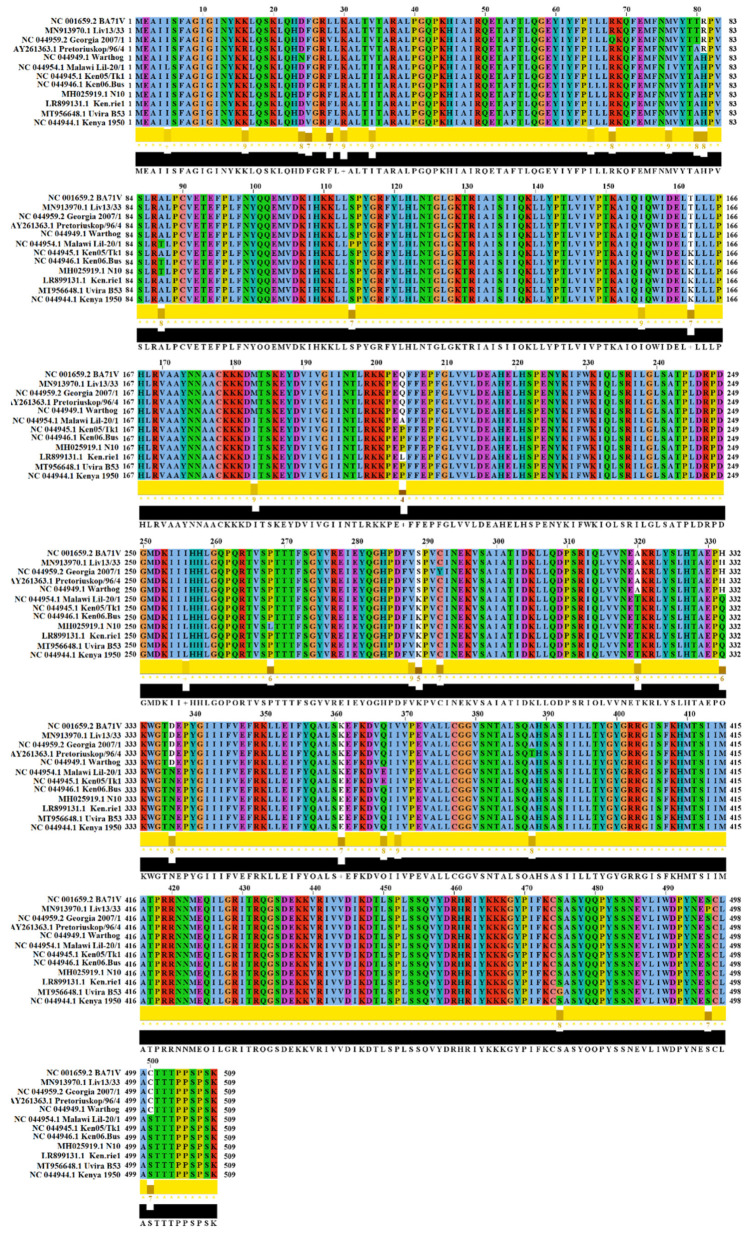
Evolution of QP509L protein among ASFV isolates. Amino acid alignment produced by the software Jalview version 2.11.1.7 showing the diversity of QP509L protein among a group of 12 representative ASFV isolates. Conservation plot scores reflect the nature of the change in specific sites. High scores are associated with changes with similar biological properties.

Along with *Q706L*, *QP509L* is one of the two RNA helicases described in ASFV [11]. Previously, others have shown the conservation of these two helicases between virulent and non-virulent isolates [11]. To acquire more insights into the evolution of the *QP509L* gene, we conducted a systematic and comprehensive evolutionary analysis, as previously described [18]. Firstly, we established a model for the analyses. For this purpose, using the *QP590L* gene from the ASFV Georgia 2007/1 isolate, we conducted a blast analysis to obtain a group of isolates representing the genetic diversity of this gene in nature: a total of 12 ASFV isolates, representing genetic groups I, II, III, VII, VIII, IX, X, and XX. To assess conservation among isolates, we conducted a pairwise analysis using the p-distance model and 100 bootstraps to support statistical confidence (p-0.05) in the analysis [19]. Overall, we found an identity between 94.49–99.86% (~96.43%) and 95.87–99.80% (97.61%) at the nucleotide and amino acid levels, respectively, showing the degree of conservation among the natural isolates. A graphic comparison of the amino acid sequences among these isolates is presented in Figure 1. No differences at the nucleotide and amino acid levels were seen among the viral isolates belonging to the pandemic Eurasian lineage (genotype II), indicating that *QP509L* is not promoting the divergence of this lineage during the pandemic.

**Figure 2:**
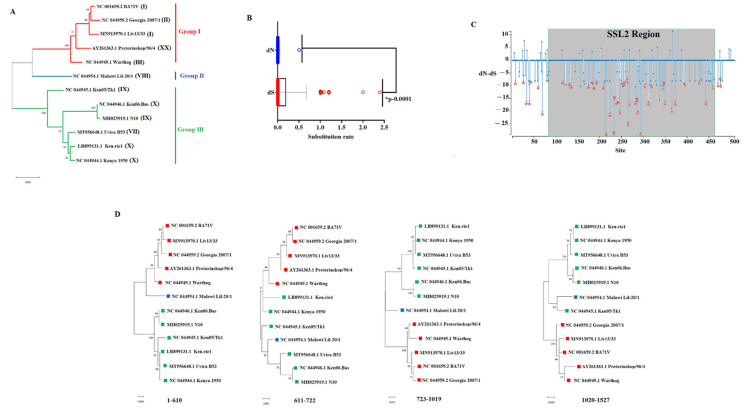
Evolutionary dynamics of the *QP509L* gene. (**A**) Phylogenetic analysis conducted by the maximum likelihood method, showing the diversity of the QP509L gene of ASFV in the field. Based on the cluster distribution, isolates were classified into three main groups (I, II, III). Numbers in the parentheses show the genetic lineage classification of different ASFV isolates based on the p72 classification. Numbers above internal branches represent bootstrap values (1000 repetitions). Phylogenetic analysis was conducted using the software MEGA 10.2.5. (**B**) Comparison between synonymous (dS) and nonsynonymous (dN) substitutions rates during the evolution of the *QP509L* gene. Significant differences between means at dS and dN were determined by the unpaired *t*-test. (**C**) Graphic representation of the ratio dN–dS at specific codon sites in the *QP509L* gene of ASFV. The region highlighted in grey represents the location of the functional region SSL2, while red asterisks represent codon sites evolving under purifying selection. Specific sites under purifying selection at the SSL2 region include: 117, 132, 135, 175, 185, 187, 193, 209, 215, 224, 233, 234, 241, 242, 246, 250, 255, 258, 264, 265, 269, 283, 285, 287, 294, 304, 312, 318, 345, 347, 349, 365, 373, 394, 428, 443, and 459. Analysis was conducted using the evolutionary algorithms FEL considering a cutoff value of *p* = 0.1. (**D**) Phylogenetic analyses showing topology incongruences among different segments where distinct breakpoints were inferred by GARD on the *QP509L* gene. Squares of different colors represent phylogenetic groups identified in Figure 2A. Numbers above internal branches represent bootstrap values (1000 repetitions). Phylogenetic analysis was conducted as described in Figure 2A.

Then, to identify the existence of potential phylogenetic groups, we conducted a phylogenetic analysis using the maximum likelihood method and the general time reversible model. Based on this analysis, we are reporting the existence of three phylogenetic groups (Figure 2A). No phylogenetic relationship was found among the previously predicted genetic groups using the p72 gene [20], showing the complex evolutionary dynamics among ASFV isolates in nature. Using the single-likelihood ancestor counting algorithm (SLAC) [21], we determined global rates of dN/dS = 0.130 along *QP509L*, indicating that purifying selection is the main force shaping the evolution of this gene in nature, a pattern consistent with our previous analyses evaluating other genes directly implicated in the replication of ASFV (A104R and E165R) [22,23]. This pattern of purifying the selection is consistent with the significant—and predicted—increased synonymous substitution rate (dS) in comparison with the nonsynonymous substitution rate (dN) (Figure 2B), indicating that synonymous mutations in the QP509L gene accumulate seven times faster than nonsynonymous mutations in nature.

No evidence of positive selection was predicted when sequences were analyzed by the algorithms fixed effects likelihood (FEL) [24] as well as the mixed effects model of evolution (MEME) [25], suggesting that different amino acid substitutions predicted among ASFV isolates (Figure 1) do not produce a significant impact on the phenotype of the *QP509L* protein. This fact is consistent with a previous publication indicating the high conservation of this protein between the virulent and attenuated isolates of ASFV [11]. Instead, FEL detected a total of 47 residues along the *QP509L* protein evolving under purifying selection, suggesting the potential relevance of these residues in the function of this protein (Figure 2C). Interestingly, 37 out of the 47 residues predicted under purifying selection appeared along the conserved SSL2 region associated with the functions of transcription, replication, recombination, and repair. The specific location of these residues is shown in Figure 2C. Conversely, the rest of the residues predicted under purifying selection (residues 8, 26, 33, 40, 41, 60, 69, 471, 476, and 477) were not associated with any specific functional region. In light of these results, we suggest that multiple identified residues under purifying selection may be used as a framework for future studies to identify the relevance of specific residues in the function of the *QP509L* protein.

Finally, evidence of recombination was detected using the genetic algorithm for recombination detection (GARD) [21]. Three breakpoints were inferred by GARD at nucleotides 610, 722, and 1019 with an average model support of 0.355, 0.851, and 0.766, respectively. These inferences were supported by an improvement in the c-AIC = 6475.6, supporting the existence of potential breakpoints when compared with the score AIC-c = 6562.2 of the models, assuming no recombination. These inferences were consistent with the phylogenetic incongruences produced at multiple segments along the *QP509L* gene (Figure 2D). This result was consistent with our previous analysis conducted on helicase *A859L* [26], indicating the relevant role of recombination during the evolution of ASFV.

### 3.2. Detection of QP509L Transcription

**Figure 3:**
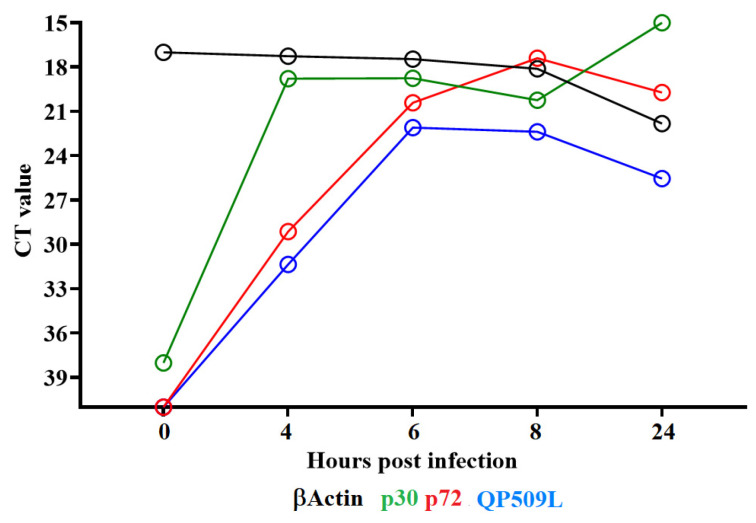
Expression profile of the *QP509L* gene of ASFV during in vitro infection of porcine macrophages. Reverse transcription followed by qPCR was used to evaluate the expression profile of the *QP509L* gene during in vitro infection at different time points, up to 24 h. As a reference for this analysis, we used qPCRs to specifically detect the expression of genes encoding ASFV proteins, p30 (early expression) and p72 (late expression). Additionally, the β-Actin gene was used as a control to evaluate the quality and levels of RNA during the infection at different time points.

Previous transcription analysis performed in Vero cells infected with the ASFV BA71V strain showed that the *QP509L* gene begins to be transcribed at 4 hours post-infection (pi) [11]. To determine the kinetics of the *QP509L* gene transcription with the field isolate ASFV-G, a time course experiment was performed to analyze the kinetics of RNA transcription in primary swine macrophages. Swine macrophages were infected (MOI = 1) and cell samples were taken at different times pi. The presence of the QP509L RNA was evaluated by RT-PCR, as described in the Material and Methods section. The patterns of transcription of the well-characterized ASFV early protein p30 (*CP204L*) and the late protein p72 (*B646L*) were used as a reference for early and late transcription profiles, respectively. The kinetics of transcription of the *QP509L* gene resembled that of the *B646L* gene, suggesting that it is transcribed as a late protein (Figure 3).

### 3.3. Development of the ASFV-G-ΔQP509L Deletion Mutant

**Figure 4:**
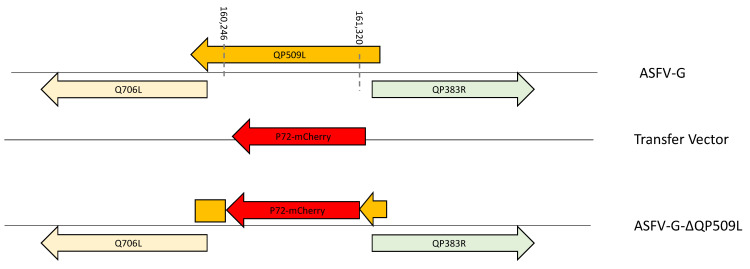
Development of ASFV-G-∆QP509L. The recombinant vector composed of the p72 promoter and an mCherry cassette, as well as the gene positions, are indicated. The homologous arms were designed to have flanking ends on both sides of the deletion/insertion cassette. The nucleotide positions of the deleted area are shown by the dashed lines. The resulting ASFV-G-∆QP509L virus is shown on the bottom, with the orange arrow representing the N-terminal portion of QP509L that remains, and the orange block representing the C-terminal portion of the QP509L that remains without a promotor.

The nucleotide and amino acid conservation of the *QP509L* gene across ASFV isolates, together with its experimentally tested RNA helicase function, would indicate that this gene may play an important role in the processes concerning virus replication and virulence in pigs. To study the potential function of the *QP509L* gene in both processes, a recombinant virus, named ASFV-G-∆QP509L, was designed by deleting the *QP509L* gene from the genome of the highly virulent ASFV Georgia 2010 isolate (ASFV-G). The *QP509L* gene was substituted with the p72mCherry∆QP509L cassette by homologous recombination with the genome of the ASFV-G [17]. The ASFV-G-∆QP509L genome has a deletion of 1075-bp, (between nucleotide positions 160,246 and 161,320), and that area of the genome was replaced by a 1226-bp cassette containing the p72mCherry construct (Figure 4). The recombinant virus was purified after several steps of limiting dilution on primary swine macrophage cell cultures.

The precision of the genomic modifications introduced in the ASFV-G-∆QP509L genome was further assessed by analyzing the full genome sequence using NGS. The obtained sequence showed the presence of the predicted 1075 nucleotide deletion, as well as the insertion of 1226 nucleotides corresponding to the p72-mCherry cassette insertion. No other significant genomic changes were identified in the ASFV-G-∆QP509L genome. The NGS analysis also showed the absence of the parental ASFV g genome, excluding a probable contaminant in the ASFV-G-∆QP509L stock.

### 3.4. Replication of ASFV-G-∆QP509L in Primary Swine Macrophages

**Figure 5:**
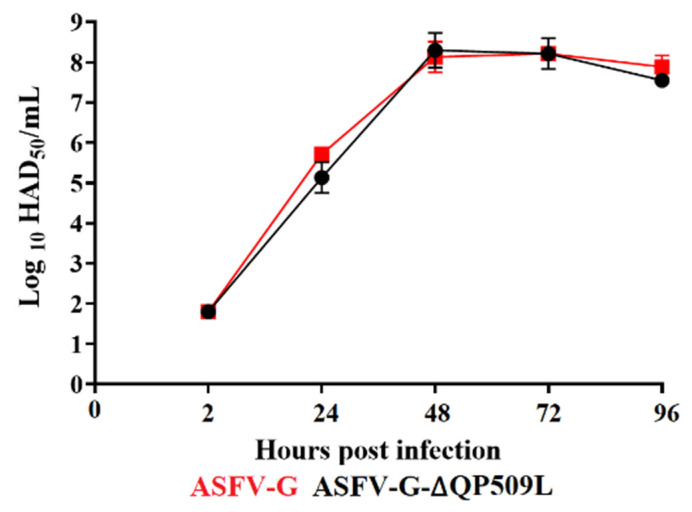
In vitro growth kinetics in primary swine macrophage cell cultures for ASFV-G-∆QP509L and parental ASFV g (MOI = 0.01). Samples were taken from three independent experiments at the indicated time points and titrated in swine macrophages. The data represent the means and standard deviations of three replicas. The sensitivity using this methodology for detecting the virus is ≥log10 1.8 HAD_50_/mL. Unpaired *t*-test with Welch correction using the two-stage step-up (Benjamini, Krieger, and Yekutieli) method was conducted to assess statistical differences in viral yields between ASFV g and ASFV-G-∆QP509L at different hours post-infection. No significant differences between the viruses were found at any of the times tested post-infection.

To evaluate the effect of deleting the *QP509L* gene in the process of ASFV g replication, the replication ability of ASFV-G-∆QP509L in primary swine macrophages was assessed and compared to that of the parental virus. A multistep growth curve was performed in swine macrophage cultures. Cell cultures were infected (MOI of 0.01) with either ASFV-G-∆QP509L or parental ASFV g, and the virus yields assessed at different times post-infection (pi) (2, 24, 48, 72, and 96 h pi). The analysis of the results indicated that ASFV-G-∆QP509L showed kinetics of replication similar to that of ASFV g (Figure 5), demonstrating that the deletion of *the QP509L* gene does not affect the replicative ability of ASFV in swine macrophages.

This result appears to contradict those showing that the siRNA treatment of Vero cells specifically affecting QP509L transcription severely affects replication of the ASFV strain BA71V [12]. As mentioned in other cases where the differences in the effects of gene deletion are studied using models based on the use of ASFV adapted to grow in cell lines, these discrepancies could be attributed to the specificities of the genome of the virus used in each experimental model. The process of adaptation of the BA71V to replicate in Vero cells involved a significant deletion of genomic areas, affecting the left and right variable regions of the virus genome and causing the loss of 11 genes belonging to the *MGF360/505* gene families [27]. Those drastic genomic changes may lead to the elimination of virus genes which could potentially be involved in the partial or complete substitution of the function of the gene under study. On the other hand, ASFV g is a field isolate that presents undetectable significant genomic alteration. Therefore, the *QP509L* gene function could be exercised by any other gene potentially possessing a vicariant function.

### 3.5. Assessment of ASFV-G-∆QP509L Virulence in Swine

**Figure 6:**
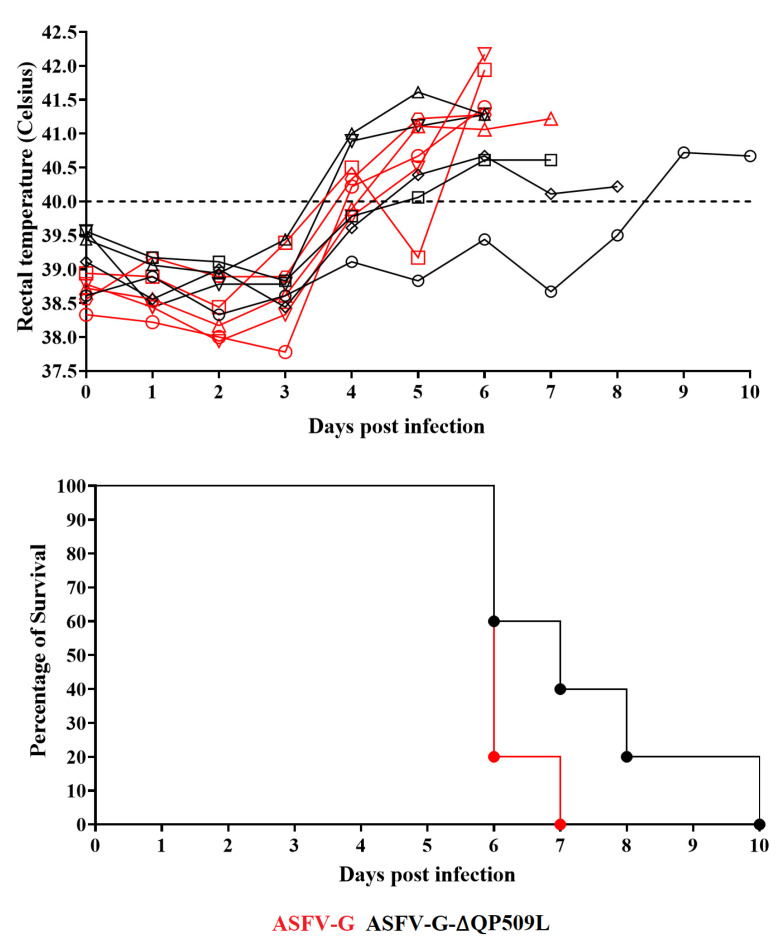
Evolution of body temperature and lethality in animals (five animals/group with each animal represented by a different shape) IM infected with 10^2^ HAD_50_ of either ASFV-G-∆QP509L or parental ASFV-G. After analyzing by both the Long-rank (Mantel-cox) and the Gehan-Breslow-Wilcoxon tests, no significant statistical differences were predicted between the different groups regarding their temperatures or lethality.

To evaluate the potential role of the *QP509L* gene in the processes of virus replication during infection, as well as in disease production in the natural host, ASFV-G-∆QP509L was used to experimentally infect domestic pigs. The experiment involved two groups of five animals weighing 35–40 kg that were infected intramuscularly (IM) with 10^2^ HAD_50_ of either ASFV-G-∆QP509L or the parental ASFV-G. The presentation of clinical signs associated with ASF was monitored during a 28-day observational period. As expected, all animals receiving the virulent ASFV g showed disease onset by day 4–5 pi with an increase in body temperature of over 40 °C. These animals quickly evolved to a severe form of clinical disease (depression, anorexia, staggering gait, diarrhea, and purple skin discoloration) with all animals needing to be euthanized by day 6–7 pi (Figure 6).

The group of animals inoculated with ASFV-G-∆QP509L also showed a lethal form of the disease, although with a slightly protracted clinical presentation in some of the animals (Figure 6). Three of the animals presented disease kinetics indistinguishable from animals inoculated with the parental virus, showing a sharp increase in body temperature by day 4–5 pi, followed by a full presentation of clinical disease with all animals needing to be euthanized on day 6–7 pi. Another animal had an onset of disease by day 5 pi, presenting a slight rise in body temperature, which evolved to a full clinical disease on the subsequent day, and it was euthanized by day 8 pi. The fifth animal remained clinically normal until day 9 pi when it presented a sharp rise in body temperature and a quick evolution of the disease, being euthanized on day 9 pi. Regardless of the existence of small differences in the time of euthanasia between the two groups of animals, it is clear that there are no strong differences in the evolution of the disease in animals inoculated with ASFV-G-∆QP509L when compared with those infected with the parental virus. Therefore, the deletion of the QP509L gene in the genome of ASFV g did not considerably influence its virulence in domestic pigs.

**Figure 7:**
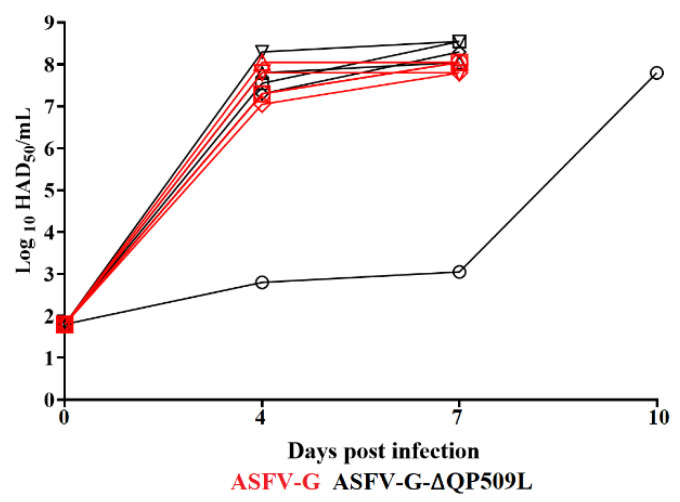
Viremia titers were detected in pigs IM inoculated with 10^2^ HAD_50_ of either ASFV-G-∆QP509L or ASFV-G. Each curve with a different shape represents individual animal titers. The sensitivity of virus detection: >log10^1.8^ TCID_50_/mL. No significant differences in the evolution of viremia were found between groups when analyzed by the unpaired *t*-test using the two-stage step-up (Benjamin, Krieger, and Yekutieli) method.

The ability of ASFV-G-∆QP509L to replicate during the experimental infection in pigs was assessed by comparing the kinetics of viremia titers to those observed in the ASFV g-infected animals (Figure 7). Animals inoculated with the virulent parental ASFV g had viremia titers ranging from 10^7^–10^8.3^ HAD_50_/mL by day 4 pi in all animals and continued at that level until the day they were euthanized. In the ASFV-G-∆QP509L-inoculated animals, viremia patterns followed the evolution of clinical disease, with four animals having viremia kinetics indistinguishable from animals inoculated with the parental virus except for the animal presenting a protracted form of the clinical disease. That animal presented low viremia titers (lower than 10^3^ HAD_50_/mL) until day 7 pi, with a robust increase by day 10 pi (around 10^7^ HAD_50_/mL), when the animal was euthanized. Therefore, viremia titers reproduced the differences in the kinetics of the presentation of the clinical signs of disease in animals inoculated with ASFV-G-∆QP509L and ASFV-G.

This report presents results that support the *QP509L* gene as a non-essential gene for the first time. When QP509L is deleted, virus replication in macrophage cell cultures is not affected. As discussed earlier, the results that were previously reported in the ASFV BA71V strain are contradictory to the results reported here. In that report, siRNA treatment decreased the transcription of the *QP509L* gene, severely affecting virus replication [23]. As in the case of other ASFV genes, where results were obtained using ASFV strains adapted to grow in cell lines, differences with results obtained using field isolates may be attributed to the specificities of the genome of the strains used. The process of adaptation of the BA71V gene to replicate in Vero cells involved a significant deletion of genomic regions affecting the left and right variable regions of the virus genome and causing the loss of 11 genes belonging to the MGF360/505 [27]. Perhaps these additional modifications in the genomic background of the parental ASFV BA71V may impede a substitution of the QP509L gene function by other virus genes, as may happen in parental ASFV-G.

Here, we also showed that the deletion of the *QP509L* gene when tested in domestic swine does not affect virus replication during infection and does not affect the level of virulence of the ASFV-G. Additional NGS analysis of the virus obtained from the blood of euthanized animals showed that only ASFV-G-ΔQP509L was present, eliminating any possibility that the disease was caused by the hypothetical presence of any residual ASFV g in the ASFV-G-ΔQP509L stock. Interestingly, in the highly virulent ASFV CN/GS/2018 strain, a recombinant virus, the deletion of the *QP509L* gene was reported to be partly attenuated in swine when inoculated IM at a dose of 10^2^ HAD_50_ [12]. It is not clear at this point if differences between these results may be produced by the inherent characteristics of the parental virus strains, although both are derivatives of the ASF Georgia 2007 strain.

Understanding the role of individual proteins in ASFV is critical, as the deletion of individual proteins has led to the discovery of experimental vaccines [2] such as the first approved commercial vaccine currently in use in Vietnam [1,6,8]. However, understanding what genes resulted in a slight reduction in virus virulence is also important, as combining gene deletions has resulted in decreased virus virulence. For example, the additional deletion of the *UK* gene added to either a single gene deletion mutant that contains *9GL* [9] or a single deletion mutant that contains CD2 [28] had the result of complete attenuation in swine, while the UK or CD2 individual gene deletions, similar to QP509L, were not able to attenuate ASFV-G. Another example occurred with the MGF (multi-gene-family) genes, wherein individual family members can be deleted without affecting ASFV pathogenesis [29,30,31] or only producing partial attenuation [32]. However, larger deletions in this region have had further attenuation [5,33], or, in some cases, the complete loss of ASFV’s ability to replicate in swine [14]. In other cases, deletions could allow the growth of continuous cell cultures [34]. These results highlight the difficulty in choosing genetic targets for deletion to attenuate current or potential future emerging strains of ASFV. Experimentally testing the effect of gene deletion in animals is the only method to actually evaluate the function of a particular gene in the process of virulence in pigs [1].

Additional deletions could be used in next-generation live attenuated vaccines or as a serological DIVA target that would allow for differentiation between an infected animal or a vaccinated animal; however, adding serological markers to current vaccines has only reduced vaccine efficacy [35]. Therefore, understanding which genes could possibly be deleted from the ASFV g genome is critical information, as the essentiality of some genes has varied between isolates. For example, EP152R [36] from the ASFV g isolate could be deleted [37] but not in previously tested isolates [38]. Identifying genetic deletions in any current or future circulating ASFV strains is critical to understand, as this information will be necessary for future rationally designed ASF vaccines in response to emerging or new strains of ASFV.

## Data Availability

Not applicable.

## Supplementary Materials

The following supporting information can be downloaded at: https://www.mdpi.com/article/10.3390/v14112548/s1, Figure S1: Coverage map.
