# Supplementary figures and images for: Deletion of an African Swine Fever Virus ATP-Dependent RNA Helicase QP509L from the Highly Virulent Georgia 2010 Strain Does Not Affect Replication or Virulence
Source: Viruses. 2022 Nov 17;14(11):2548. doi: 10.3390/v14112548 (PMC9694930; doi:10.3390/v14112548)

Supplementary Figure S1: Coverage map

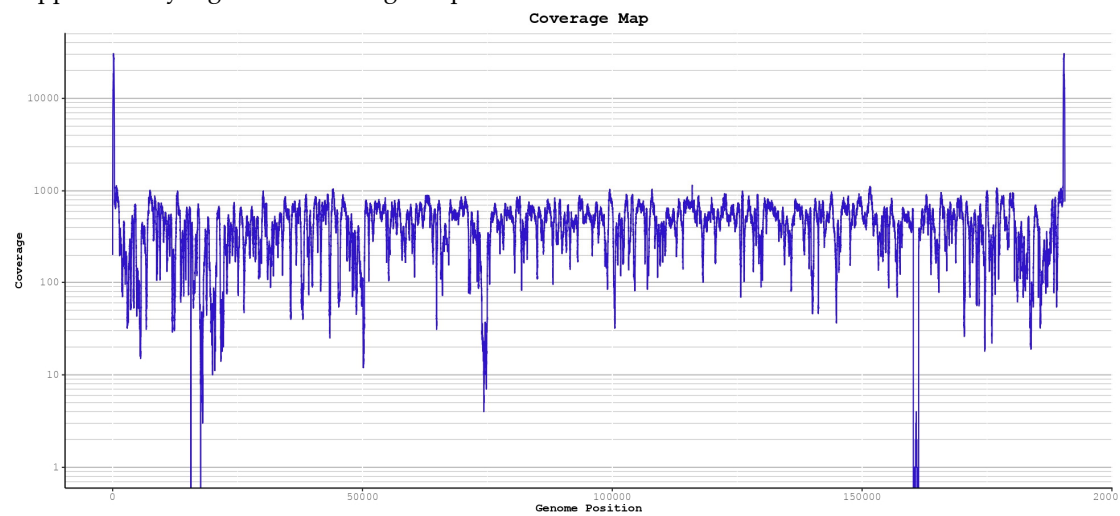

Supplement: Supplementary file 1 [file viruses-14-02548-s001.zip › viruses-1952361-supplementary.pdf]
